# Posttranslational Modification in Bone Homeostasis and Osteoporosis

**DOI:** 10.1002/mco2.70159

**Published:** 2025-04-01

**Authors:** Yuzhe Lin, Shide Jiang, Yuming Yao, Hengzhen Li, Hongfu Jin, Guang Yang, Bingzhou Ji, Yusheng Li

**Affiliations:** ^1^ Department of Orthopedics Xiangya Hospital Central South University Changsha China; ^2^ Xiangya School of Medicine Central South University Changsha China; ^3^ The Central Hospital of Yongzhou Yongzhou China; ^4^ National Clinical Research Center for Geriatric Disorders Xiangya Hospital Central South University Changsha China

**Keywords:** bone formation, bone resorption, osteoporosis, posttranslational modification

## Abstract

Bone is responsible for providing mechanical protection, attachment sites for muscles, hematopoiesis micssroenvironment, and maintaining balance between calcium and phosphorate. As a highly active and dynamically regulated organ, the balance between formation and resorption of bone is crucial in bone development, damaged bone repair, and mineral homeostasis, while dysregulation in bone remodeling impairs bone structure and strength, leading to deficiency in bone function and skeletal disorder, such as osteoporosis. Osteoporosis refers to compromised bone mass and higher susceptibility of fracture, resulting from several risk factors deteriorating the balanced system between osteoblast‐mediated bone formation and osteoclast‐mediated bone resorption. This balanced system is strictly regulated by translational modification, such as phosphorylation, methylation, acetylation, ubiquitination, sumoylation, glycosylation, ADP‐ribosylation, S‐palmitoylation, citrullination, and so on. This review specifically describes the updating researches concerning bone formation and bone resorption mediated by posttranslational modification. We highlight dysregulated posttranslational modification in osteoblast and osteoclast differentiation. We also emphasize involvement of posttranslational modification in osteoporosis development, so as to elucidate the underlying molecular basis of osteoporosis. Then, we point out translational potential of PTMs as therapeutic targets. This review will deepen our understanding between posttranslational modification and osteoporosis, and identify novel targets for clinical treatment and identify future directions.

## Graphical abstract

This review summarizes the updating researches concerning bone formation and bone resorption mediated by different types of posttranslational modification. We highlight dysregulated posttranslational modification in osteoblast and osteoclast differentiation. We then emphasize involvement of posttranslational modification in osteoporosis development, so as to elucidate the underlying molecular basis of osteoporosis. We also suggest translational potential of PTMs as therapeutic targets

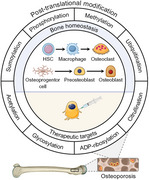

## 1 Introduction

Bones of the musculoskeletal system is continuously remodeled through bone formation and bone resorption, serving as mechanical scaffold for muscle attachment and locomotion. Bone also provides a venue for hematopoiesis to happen within bone marrow, and calcium and phosphate to be reserved [1]. As a highly dynamic tissue, the biological function of bone is also exhibited by the capacity to remodel and regenerate, which requires an intricate balance between bone formation and bone resorption.

The two main cell types involved in bone metabolism are osteoblasts and osteoclasts, which is responsible for an osteon to be formed and damaged bone to be removed, respectively. Osteoblast and osteoclast derive from different cell lineage. The osteoblast, which originates from mesenchymal stem cells (MSCs), undergoes sequential stages of differentiation: osteoprogenitor cells, preosteoblast, and osteoblast [2]. In the commitment to osteoblast, several transcriptional factors act sequentially in the three phases: SOX9, which potentiates differentiation into osteoprogenitor cells; Runt‐related transcription factor 2 (RUNX2) responsible for specification of preosteoblast; and osterix (OSX or SP7) determines the differentiating fate of osteoblast [3]. Other transcriptional factors have been identified to be implicated in specification of skeletal lineage, such as the activator protein 1 (AP‐1) and ATF4 [4, 5]. Osteoclast arises from hematopoietic lineage. And the transcriptional factor essential for osteoclast differentiation is RANKL, which encourages osteoclast precursors to be specialized into osteoclasts [6]. Bone remodeling and bone homeostasis is strictly under the regulation of osteoblast‐mediated bone formation and osteoclast‐mediated bone resorption, and imbalance between osteoblastogenesis and osteoclastogenesis resulted from dysregulation of related signaling pathways or certain transcriptional factors could affect bone remodeling, which potentially leads to osteoporosis (OP).

OP is a skeletal disorder featured by decreased bone density and increased risk of bone fracture. Susceptibility of fracture and alteration in demographic structure exert enormous burden on individual and heal care worldwide. Several factors are documented to increase the risk of OP, which include but not limited to aging, hormone disorders, and lack of mechanical stimulus [7]. As mentioned above, the underlying molecular mechanism accounting for OP is imbalance between bone resorption and bone formation induced by abnormality in osteoblast and osteoclast function or number. One of mechanism contributing to regulated osteoblastogenesis and osteoclastogenesis is protein posttranslational modification (PTM), which refers to the dynamic and reversible decoration of target protein with certain chemical moieties, peptides, or sugars. PTMs have a profound impact on protein diversity, as it affects protein's physicochenmical characteristics, such as protein stability, hydrophobicity, protein degradation, and protein–protein interaction, which leads to alteration in the abundance and function of proteins [8]. A wide spectrum of modified protein is thus engaged in multiple signaling pathways and cellular processes, making dysregulation of PTMs being implicated in disruption of bone homeostasis and development of OP. More than 600 kinds of PTMs are documented, such as phosphorylation, acetylation, and ubiquitination [9]. Importance has been attached to the significant role of PTMs in maintaining bone homeostasis and OP. Current studies mainly focus on the underlying molecular mechanism of each type of PTMs involved in bone homeostasis and OP. However, less is summarized about the involvement and interaction of different types of PTMs in coordinating bone resorption and formation, as well as OP development.

**FIGURE 1:**
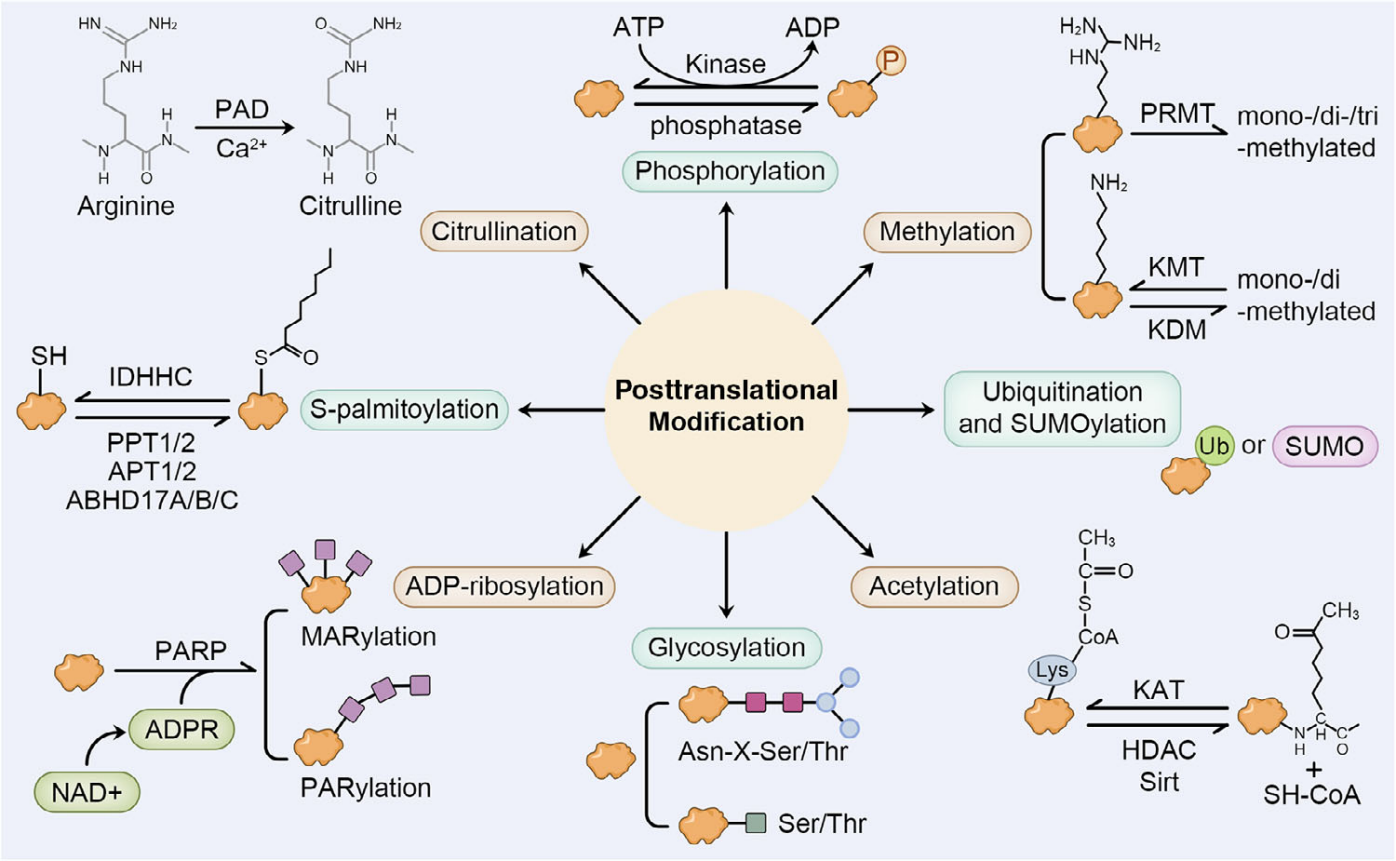
Posttranslational modification in bone homeostasis and osteoporosis. Posttranslational modification is the chemical modification of multiple amino acid residues after protein translation. What are circled in orange refers to substrate proteins. The purple square stands for ribose. Green circle with “Ub” stands for ubiquitin, while SUMO refers to small ubiquitin‐like modifier.

In this review, we will first introduce the basics of bone homeostasis, OP, and PTM, respectively, then discuss the latest researches concerning how each common type of PTMs (phosphorylation, methylation, acetylation, ubiquitination, sumoylation, glycosylation, ADP‐ribosylation, S‐palmitoylation, and citrullination) is involved in correlative signaling pathways, cell differentiation, metabolism, and other cellular processes related to bone homeostasis and OP progress, respectively. We also highlight the potential therapeutic roles of PTMs in OP treatment, and suggest current research gaps, with the aim of better understanding in biological function of PTMs in OP (Figure 1).

## 2 Overview

### 2.1 Bone Homeostasis and OP

Bone homeostasis refers to the balance between bone resorption and bone formation [10], which is mainly maintained by osteoblasts and osteoclasts. Derived from the skeletal lineage, the osteoblasts are essential in bone development and bone growth through the secretion of extracellular matrix, including type I collagen, osteopontin, alkaline phosphatase (ALP), and so on. Originated from the skeletal lineage, osteoprogenitors are specified into preosteoblasts and osteoblasts under the regulation of several indispensable transcriptional factors, such as runt‐related transcription factor 2 (Runx2) and osterix (Osx) [11, 12]. Along with other signaling pathways that is discussed in the following paragraphs, these transcriptional factors signify the commitment to osteoblasts, which further undergo three different cell fates, osteocytes, bone‐ling cells, or apoptosis [13, 14]. Embedded into the osteoid matrix, osteocytes are the most abundant cell types in mature mineralized bones and is involved in maintaining bone homeostasis [15]. For example, osteocytes have a dual effect on bone formation through production of sclerostin and insulin‐like growth factor (IFG)‐1 respectively [16, 17]. Osteoclasts, responsible for bone resorption, are derived from hematopoietic stem cells [18]. By secreting acids and proteases, such as cathepsin K (CTSK), osteoclasts degrade extracellular proteins and collagen in precise positions [19]. Two significant transcriptional factors mediating commitment to osteoclasts are colony‐stimulating factor and RANKL, which facilitate osteoclast precursors proliferation and direct osteoclast precursor differentiation into osteoclasts, respectively [20, 21]. Under strictly controlled programs, balance between osteoblasts and osteoclasts regulates appropriate bone resorption and bone formation, and thus ensuring appropriate bone mass. However, dysregulation of this process leads to bone loss, which consequently presents mechanical and structural bony pathologies, such as OP [22, 23].

OP is a common disease affecting more than 200 million people worldwide, which imposes huge burdens on public health system [24]. Featured by loss in bone mass, density, quality, and high susceptibility to fractures, OP is considered as a result of dysregulation of multiple cellular processes involved in bone homeostasis [22]. From the perspective of etiology, OP is categorized into primary and secondary OP [25]. Primary OP accounts for over 95%, which mainly covers involutional and idiopathic OP that have an influence usually on the young and the senior respectively [25]. Occupying a relatively small proportion, secondary OP is caused by medication or considered as a consequence of other diseases [26]. On cellular level, OP is linked to unbalanced bone resorption and bone formation, which indicated OP is attributed to the differentiating tendency to adipogenesis rather than osteogenesis [22]. An intriguing instance would be that upregulating RNAK‐L causes OP, while suppressing RANKL signaling is supposed to be a therapeutic target of bone mass loss in OP [27]. Therefore, emerging researches on regulatory mechanism of bone homeostasis is now providing novel insights in better understanding of OP occurrence and development [28].

### 2.2 Posttranslational Modification

PTM is a form of protein folding, subcellular localization, stability, and activity regulation through the chemical modification of various amino acid residues after protein translation [29]. PTMs enable proteins to be implicated in a wider range of cellular processes with only 20 canonical amino acids. Given their expansive variation in the substrate amino acid, chemical groups, and metabolic resources, categorization of PTMs spans a wide spectrum, while some commonly discussed PTMs mainly include phosphorylation, methylation, ubiquitination, acylation, and so on [30]. On the contrary, the biological functions of some other forms of PTMs in bone homeostasis, such as glycosylation, ADP‐ribosylation, S‐palmitoylation, and citrullination, are relatively scantly investigated.

As a covalent modification after protein synthesis is under the regulation of specific enzymes, which catalyze recognition of protein substrate, addition of relevant functional chemical groups, and removal of corresponding functional groups. The intricate regulatory map of each PTM has a profound effect on protein abundance, and thus affecting protein–protein interactions and signal transduction [31]. And abnormalities in their cellular levels or enzymatic activities under pathologic conditions are implicated in multiple disease progress, for instance, remodeling tumor microenvironment [32], exaggerating inflammation response [33], and triggering profibrotic regulators [34]. Similarly, dysregulation of PTMs concerning osteogenesis and osteoclastogenesis affects bone formation and resorption, disrupting bone homeostasis that could lead to pathological state of bones, such as OP. Therefore, elucidating underlying mechanisms of osteogenesis and osteoclastogenesis on the posttranslational level is of great significance to deepen our understanding of bone homeostasis, and thus filtering potential drug candidates for OP.

## 3 Phosphorylation in Signaling Pathways

Phosphorylation is one of the most common PTMs regulated by kinases and phosphatases to add and remove a terminal phosphate group to a polar group of amino acids, respectively. The deposition of a phosphate group, which mostly occurs on serine and threonine residues, usually endows the modified proteins with hydrophilicity and polarity to bind to other proteins or molecules [35]. Therefore, phosphorylation is considered as an essential regulation of cellular proteins and is thus implicated in various biological processes, including signal transduction, cell cycle, and cell differentiation [36].

**FIGURE 2:**
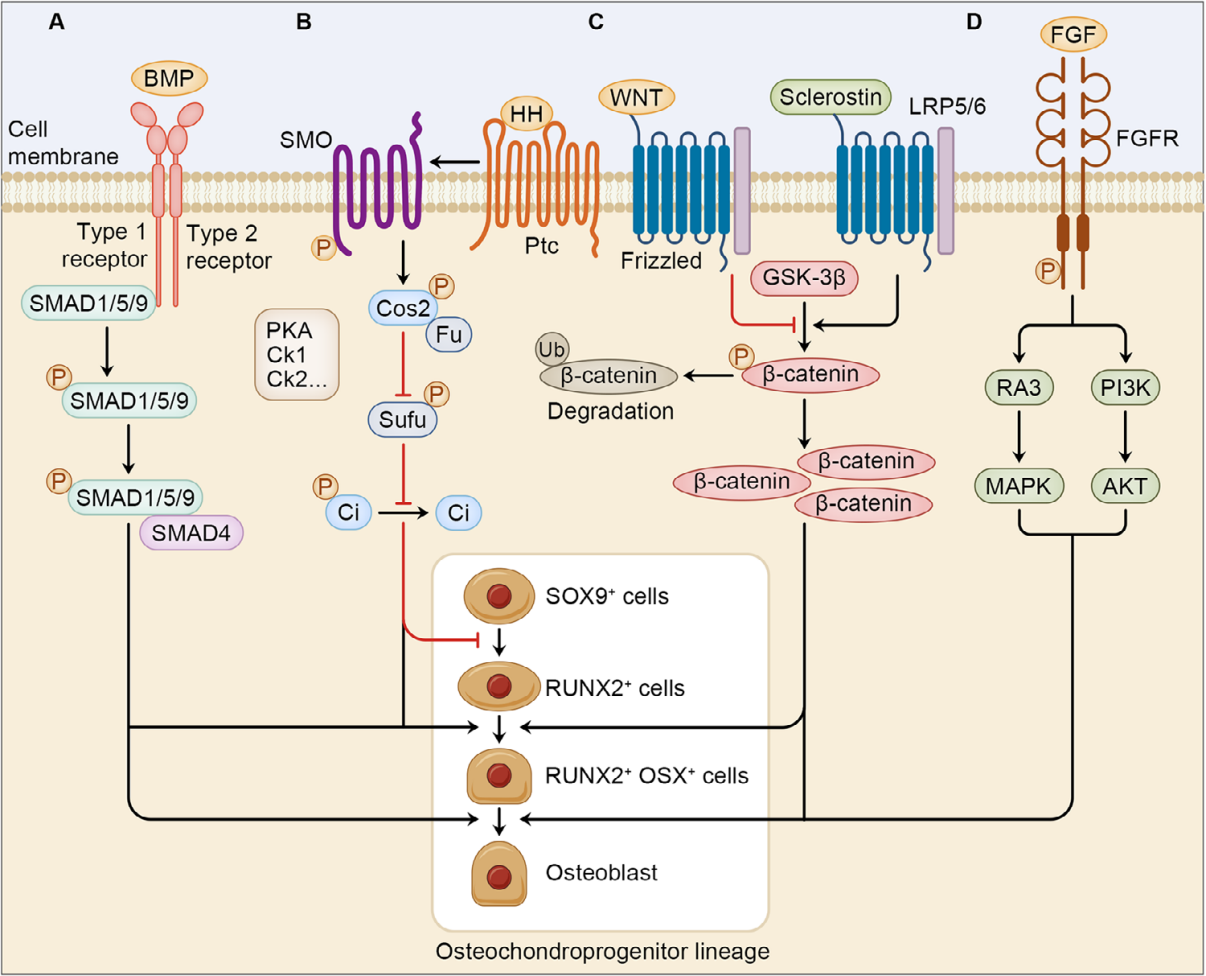
Phosphorylation in signaling pathways involved in osteoblast differentiation. *Note*: In the regulation of osteogenesis, phosphorylation is implicated in some pathways indispensable for osteoblastogenesis. (A) Smad proteins are phosphorylated to bind to SMAD4, which further signifies commitment to osteoblast. (B) SMO is phosphorylated after HH ligand binds to Ptc, which leads to Cos2 phosphorylation and Ci dephosphorylation that causes elevated transcription of RUNX2. (C) Phosphorylation of GSK‐3β is suppressed when Frizzled is activated by WNT ligand, which results in increased RUNX2 and OSX expression and promotes osteoblast differentiation. Phosphorylation of GSK‐3β is conversely promoted by sclerostin that inhibits commitment to osteoblast. (D) FGFR is phosphorylated after interaction with FGF, which leads to activation of several signaling pathways and osteoblastogenesis.

In the process of osteogenesis and osteoclastogenesis, several signaling pathways are considered indispensable for their involvement in the response to external stimulus, which mainly include bone morphogenetic protein (BMP) signaling, Hedgehog (Hh) signaling, Wnt signaling, and fibroblast growth factor (FGF) signaling. Phosphorylation plays a vital role in regulating typical signaling pathways and transcriptional factors of osteoblast and osteoclast differentiation. Therefore, alterations in a protein phosphate site causes dysregulated signaling pathways and thus disrupting bone formation and resorption. In this section, we mainly focus on the signaling pathways that are crucial for osteogenesis with the involvement of phosphorylation (Figure 2).

### 3.1 BMP Signaling

As a member of the transforming growth factor‐beta superfamily, BMPs transduce signals in a SMAD‐dependent manner, which finally enters the nucleus to regulate target gene expression [37]. In response to the BMP signaling, BMP receptor (BMPR) on the membrane forms a heteromeric complex consisting of a type 1 receptor and a type 2 receptor, both of which belong to the serine/threonine kinase family [38]. This combination allows the type 1 receptor to be phosphorylated by the type 2 receptor. The phosphorylated Smad proteins thus initiate intracellular signaling transduction [39]. The activated Smad proteins, usually the SMAD1, SMAD5, and SMAD9, bind to SMAD4, and is then transfected into nucleus to regulate downstream gene expression that is significantly implicated in osteogenesis, including Runx2 and Osx [38, 40]. Therefore, intricate phosphorylation level involved in BMP signaling pathway is crucial in cell differentiation, as depleted Runx2 in mice leads to osteoblast reduction and reduced bone formation [41, 42]. Notably, therapeutic treatment targeting BMP signaling has been confirmed effective in relevant diseases, for example, recombinant human BMP2 and BMP7 are now clinically available in OP and other bone deficiencies [43]. However, considering low specificity of phosphorylation regulators, further investigation is needed concerning targeting phosphorylase as drug candidates in mediating osteogenesis.

### 3.2 Hh Signaling

The past decades have witnessed emerging evidence recognizing Hh signaling as an evolutionarily conserved pathway in determining the anterior–posterior body axis, limb development, and bone homeostasis [44]. The transduction of Hh signaling is dependent on the two receptor proteins, Smoothened (Smo) and Patched (Ptc). Smo is a seven transmembrane protein with serine and therenine residues at the C‐terminus to be phosphorylated by kinases. In the absence of Hh protein, Smo is inhibited by Ptc. This deactivation of Smo is reversed once the Hh ligand interacts with Ptc to initiate the intracellular signaling, followed by the phosphorylation of Smo catalyzed by several kinases, including casein kinase 1 (CK1), casein kinase 2 (CK2), and protein kinase A (PKA) [45]. Then, Costal 2 (Cos2) is recruited at Smo and phosphorylated by Fused (Fu), while Cubitus interruptus (Ci) that used to be phosphorylated by Cos2 complex is released and therefore relocates into the nucleus to regulate target gene expression.

The function of Hh signaling in bone homeostasis spans a wide spectrum, and a prominent example would be its role in endochondral ossification, as deficiency in IHH signaling leads to impaired osteoblast differentiation in mouse SSCs [46]. Therefore, Hh signaling exhibits significance with its implication in bone repair. In fracture repair, Hh signaling functions as a downstream pathway triggered by parathyroid hormone (PTH). Following the binding of PTH to PTHR1, cAMP level is elevated to induce the activation of PKA to phosphorylate CREB that upregulates IHH gene expression [47]. However, it is noticeable that Shh signaling is not a requisite in osteoblast differentiation, especially when prompted by fibroblast‐released factors that upregulated the downstream proteins of Shh [48]. Given that the biological functions of phosphorylation involved in Hh signaling range from affecting osteoblast differentiation directly to responding to PTH, relevant phosphorylates is suggested to be therapeutic targets to improve bone homeostasis, though related researches are still lacking.

### 3.3 Wnt Signaling

Wnt signaling pathway is divided into canonical and noncanonical Wnt signaling pathways, with the former one having an established link to osteogenesis. Wnt signaling is potentiated by the binding between Wnt ligand and receptor Frizzled and low‐density lipoprotein receptor‐related protein 5 (LRP5) or LRP6. Activation of Wnt signaling inhibits lycogen synthase kinase‐3β (GSK‐3β)‐mediated phosphorylation of β‐catenin and resulted in β‐catenin accumulation. Accumulated β‐catenin is further trafficked into nucleus for transcriptional activity. The biological function of Wnt signaling has been broadly elucidated, as gain‐of‐function and loss‐of‐function of LRP5 upregulates or downregulates bone mass through regulation of osteoblastogenesis [49, 50].

Notably, Wnt signaling can be suppressed by sclerostin. Sclerostin is an osteocyte‐excreted protein encoded by SOST gene. In the absence of Wnt, sclerostin inhibits Wnt signaling pathway through interacting with LRP5/6, leading to phosphorylation, ubiquitination, and degradation of cytosolic β‐catenin. The expression of sclerostin within osteocytes is promoted by PTH, which binds to PTH‐related protein (PTHrP). Thus, salt‐inducible kinase (SIK)‐mediated histone deacetylase (HDAC)4/5 phosphorylation is inhibited to impede MEF2‐induced sclerostin transcription [51]. Therefore, suppression of SIK leads to decrease in sclerostin excretion and amelioration of OP [52]. In general, dysregulation of Wnt signaling disrupts osteoblast differentiation and accelerates OP development, Therefore, deepening understanding in Wnt signaling that is responsible for osteogenesis informs correlated pathological processes, for example, targeting SIK for deletion is suggested a therapeutic target to attenuate OP.

### 3.4 FGF Signaling

FGFs is composed of a group of signaling molecules implicated in human development, including neural development, skeletal development, and limb development [53, 54]. By binding to the FGF receptors (FGFRs), the tyrosine residue of FGFRs is phosphorylated to activate several signaling pathways, such as MAPK pathway and PI3K pathway [55].

Emerging evidence has indicated the correlation between FGF/FGFR signaling and bone formation. FGFs either positively or negatively regulate bone development. For example, knockout of FGF2, FGF8, and FGF10 induce defective limb development and lower bone mass in mice [56, 57]. The FGF23, excreted by osteoblasts and osteocytes, suppresses osteogenic differentiation of BMSCs. By activating FGFR3, FGF23 induces phosphorylation of ERK to inhibit osteogenesis in chicken [58]. Another mechanism by which FGF signaling negatively regulates bone elongation is its interaction with natriuretic peptide receptor 2 (NPR2) signaling. Under the regulation of FGFR3 activated by FGF18, NPR2 is dephosphorylated and deactivated, resulting in decreased cGMP‐reliant PRKG2 that contributes to and enhanced bone elongation [59].

Also, the dysregulation of the membrane receptors for FGFs, FGFRs, exhibit abnormalities in suture development and osteogenesis. For instance, the activating mutation of FGFR1‐3, whereby FGFR1‐2 are the primary subtypes in osteoblasts, advances cranial suture ossification [60], while the inactivation of Fgfr1 in mice enhances osteoblast proliferation. Notably, the expressing level of FGFR1 varies with the differentiation stages of osteoblast, which is specifically elevated in pace with osteoblastogenesis. This alteration in FGFR1 level is supported by the results that suppression of FGFR1 leads to suppressed or prompted osteoblast differentiation in osteoprogenitor and osteoblast respectively [61]. Therefore, the biological role of FGF signaling extends from promoting bone formation and elongation, cranial suture ossification, to signifying differentiating commitment to osteoblasts, and is thus considered potential therapeutic target in activating bone repair. However, preclinical and clinical experiments are still lacking. Future advancements should address its applicability as therapeutic targets.

## 4 Methylation in Bone Homeostasis and OP

Protein methylation is a significant PTM involved in multiple biological processes, such as DNA repair, epigenetic regulation, and so on. Past decades have witnessed emerging evidence increasing our knowledge concerning protein methylation and thousands of methylation sites being identified [62]. Catalyzed by the methyltransferase enzymes, the methyl group is transferred from S‐adenosylmethionine (SAM) to protein residue. Protein residue being most frequently methylated is often arginine and lysine [63, 64]. According to the abundance of hydrogen groups locating at the targeting amino group, arginine can be mono‐, di‐, while lysine can be further tri‐methylated.

### 4.1 Arginine Methylation

Arginine methylation is mediated by protein arginine methyltransferases (PRMTs), which transfer methyl groups from SAM to guanidino groups on arginine side chain [65]. Based on their catalytic activities, the nine PRMTs can be categorized into three groups, including ω‐N^G^‐monomethylarginine (PRMT1‐4, PRMT 6, and PRMT8), ω‐N^G^‐dimethylarginine (PRMT5 and PRMT9), and N′G‐dimethylarginine (PRMT7). Emerging evidence have revealed their roles in mediating gene transcription, protein degradation, and determining cell fate [66], while in bone development and pathogenesis of OP, PRMTs are involved in both osteoblastogenesis and osteoclastogenesis via the regulation of glucose metabolism and key transcription factors.

In the process of osteogenesis, coactivator‐associated arginine methyltransferase 1 (CARM1), also known as PRMT4, facilitates bone formation by affecting glucose metabolism. Mechanistically, CARM1 catalyzed protein phosphatase 1 catalytic subunit alpha (PPP1CA) methylation at R23, leading to reduction in PPP1CA dephosphorylation level and increase in AKT–Thr450 and AMPK–Thr172 phosphorylation that activates phosphofructokinase‐1 (PFK1) and pructose‐2,6‐biphophatase3 (PFKFB3) in the promotion of glycolysis [67]. In the process of osteoclastogenesis, PRMT1 is positively correlated with osteoclastogenesis through the interaction with p65 and the activation of NF‐κB signaling pathway. Such RNAKL‐dependent PRMT1 expression can be suppressed by estrogen, which indicates its promotional effect in postmenopausal OP [68]. Another intriguing example is PRMT5, which dimethylates arginine with PRMT7, catalyzes H3R8 and H4R3 methylation at C‐X‐C motif ligand (CXCL) 10 and RSAD2 promoter that leads to osteoclastogenesis promotion [69]. Therefore, clinical bone diseases offer novel perspectives on bone homeostasis, and arginine methyltransferases or demethylates are suggested potential therapeutic targets, for their involvement in osteoclast differentiation that exerts a profound effect on OP development (Table 1).

**TABLE 1:** Methylation and demethylation in bone homeostasis.

	Enzymes	Gene target (amino acid residue/histone target site)	Function	References
Arginine methyltransferases	PRMT1	P65	Activates NF‐κB signaling and osteoclastogenesis	[68]
	PRMT4 (CARM1)	PPP1CA (R23)	Activates AKT and AMPK signaling, promotes glycolysis, inhibits osteoclastogenesis	[67]
	PRMT6	CXCL10 and RSAD2 (H3R8me2 and H4R3me2)	Promotes osteoclastogenesis	[69]
Lysine transferases	SETD2	LBP (H3K36me3)	Promotes osteogenesis	[70]
	SETDB1	OTX2 (H3K9me3)	Promotes osteogenesis through Wnt/β‐catenin and BMP–Smad pathways	[71]
	G9a	Runx2 (H3K9me2)	Promotes cranial development	[72]
	EZH2	Foxc1	Inhibits NF‐κB signaling pathway	[73]
	Dpy30	NFATc1 (H3K4)	Promotes osteoclast differentiation	[74]
Lysine demethyltransferases	KDM4B	Runx2 (H3K9me2)	Promotes osteogenesis	[72]
	KDM4A	SFRP4 (H3K9me3)	Inhibits osteogenesis via Wnt signaling inhibition	[75]
	KDM7A	C/EBPα, Sfrp1 (H3K9me2 and H3K28me2)	Suppresses osteoclastogenesis through Wnt signaling inhibition	[76]
	KDM6A (UTX)	DKK, DMP1 (H3K27me3)	Promotes osteogenesis	[77]
	JMJD3	EphB4, RASSF5 (H3K27me3)	Inhibits osteoclastogenesis, Promotes osteoblast apoptosis	[78, 79]
	LSD1	Runx2 (H3K4me2, H3K4me3)	Inhibits osteogenic differentiation	[80]

Abbreviations: CXCL, C‐X‐C ligand; C/EBPα, CCAAT/enhancer binding protein α; DKK, Dickkopf; DMP, Dentin matrix protein; EZH2, enhancer of zeste homolog2; Foxc1, Forkhead box C1; JMJD, Jumonji domain‐containing; KDM, lysine demethylases; LSD, lysine‐specific demethylase 1; LBP, lipopolysaccharide‐binding protein; NFATc1, nuclear factor of activated T‐cells cytoplasmic 1; PPP1CA, protein phosphatase 1 catalytic subunit alpha; RASSF, Ras association domain family; SETD, SET‐domain‐containing; SETDB, SER domain bifurcated; SFRP4, secreted frizzled‐related protein 4; UTX, ubiquitously transcribed tetratricopeptide repeat.

### 4.2 Lysine Methylation

As a relatively well‐researched PTM, lysine methylation is dynamically and reversibly regulated lysine methylatransferases and lysine demethylases (KDMs). The biological function of lysine methylation spans a wide spectrum as each methylation site on histones and methylation state exerts different regulatory impact on surrounding gene expression [81]. Therefore, its biological role extends from determining cell fate of BMSCs to intercellular interactions between osteoblast and osteoclast, while abnormal expression of its catalytic regulators leads to imbalance between bone formation and bone resorption, and causes deterioration in OP. For example, in aged mBMSCs, decrease in SET‐domain‐containing 2 (SETD2)‐mediated H3K36 trimethylation at regulatory region of lipopolysaccharide‐binding protein (LBP) results in LBP deficiency, which impairs osteogenesis and commit to adipogenesis, leading to age‐related OP [70]. Except for histone methylation, lysine methylation occurring at nonhistone proteins is also involved in osteogenesis by affecting protein functions. Evidence has shown that the level catalytic regulators of lysine methylation in OP patients differs from the normal, while experiments have indicated their indispensable role in bone homeostasis by affecting both osteoblast and osteoclast differentiation. In the following sections, the biological functions of lysine methylation will be discussed in detail according to the modification sites.

#### 4.2.1 H3K9

Depending on the targeting protein, lysine methylation occurring at H3K9 could have either promotive or suppressive impact on osteogenic differentiation. In the promotion of osteogenesis, the crucial role of SER domain bifurcated 1 (SETDB1) has been indicated by expanding evidence [82]. In OP patients, SETDB1 expression significantly decreases, while upregulating SETDB1 triggers osteogenic differentiation. SETDB1 targets OTX2 promoter for H3K9me3 modification, resulting in OTX2 suppression and consequent activation of Wnt/β‐catenin and BMP–Smad pathways [71]. In cranial bone cells, G9a, a methyltransferase modulating H3K9me2 modification, plays a vital role in mice cranial development through binding to promoter region of Runx2. A similar manner is seen in hBMSCs cells, where JMJD2B, also known as lysine demethylase 4B (KDM4B), binds to promoter regions of Runx2 for H3K9me2 demethylation, enhances RUNX2 expression that facilitate osteogenesis [72].

Opposing H3K9me3 modification, KDM4A, is capable of interacting with secreted frizzled‐related protein 4 (Sfrp4) promoter, promoting SFRP4 expression that causes Wnt signaling inactivation and osteogenesis inhibition in MSCs^75^. Another demethylase also inhibiting osteogenic differentiation is KDM7A, which demethylates both H3K9me2 and H3K28me2 at both CCAAT/enhancer binding protein α (C/EBPα) and Sfrp1 promoters, leading to Wnt signaling suppression that stimulates adipogenic differentiation and blocks osteogenesis [76].

#### 4.2.2 H3K27

Trimethylation at H3K27 plays a crucial role in both bone formation and resorption. In the promotion of osteocyte differentiation, Utx (ubiquitously transcribed tetratricopeptide repeat, X chromosome, Kdm6a), a histone H3K27 demethylase, interacts with regulatory regions of several osteocyte genes, including but not limited to Dkk1, Dmp1 [77]. In the regulation of osteoclastogenesis, another demethylate, Jumonji domain‐containing 3 (Jmjd3), plays a pivotal role in intercellular communication between osteoblast and osteoclast through EphrinB2–EphB4 signaling. Jmjd3 inhibits osteoclastogenesis by demethylating EphB4 promoter region in osteoblast, thus activating EphrinB2 ligand within osteoclast and repressing osteoclast differentiation [78, 83].

Trimethylation at H3K27 opposed by Jmjd3 also exerts an inhibitory effect on bone formation, as it increases Ras association domain family (RASSF5) expression that promotes osteoblast apoptosis [79]. While by targeting Foxc1 for H3K27 methylation, enhancer of zeste homolog2 (EZH2) suppresses osteoclastogenesis through NF‐κB signaling pathway inactivation [73].

#### 4.2.3 H3K4

Evidence have shown that demethylation or trimethylation at H3K4 controls both osteoblast and osteoclast differentiation. In MSCs, lysine‐specific demethylase 1 (LSD1) targets binding site of Runx2 to regulate its chromatin status, causing impairment in osteoblast differentiation and bone formation [80]. On the other side, the inhibitory impact on osteoclastogenesis mediated by H3K4 methylation can be illustrated by Dpy30, as is promotes NFATc1 methylation, which serves as a transcription factor of osteoclast differentiation [74]. Therefore, deepening our understanding lysine methylation responsible for bone homeostasis provides therapeutic targets for OP. However, relevant preclinical and clinical experiments are to be completed.

## 5 Acetylation in Bone Homeostasis and OP

Emerging as a widespread and biologically significant PTM, acetylation has been broadly researched for its impact on various cellular processes and potential as disease biomarkers [84]. Acetylation is reversibly regulated by acetyltransferases (KATs) and deacetyltransferases, which add or remove an acetyl moiety from acetyl‐CoA to Lys residues on substrate proteins respectively. Similar to protein methylation, the biological functions of acetylation become complicated, as its substrate protein include both histone and nonhistone proteins. For example, acetylation of histone residues is considered to promote the dissociation of DNA and histone and the recruitment of reader proteins, so as to active specific gene transcription [85]. And acetylation of nonhistone proteins has been shown to influence protein interaction and structural stability [86]. In this section, we will discuss the molecular impact of both histone and nonhistone acetylation on skeletal lineage, as well as its association with OP progression (Table 2).

**TABLE 2:** Histone acetylation and nonhistone acetylation in bone.

Cell type	Enzymes	Target gene/substrate protein	Impacts	References
BMSCs	KAT2B	SRSF1	Promotes osteogenic differentiation	[87]
MSCs and preosteoblasts	KAT8	RUNX2, OSX	Promotes osteogenesis	[88]
Primary osteoblasts	CBP/P300	RUNX2	Promotes osteogenesis	[89]
BMSCs	GCN5	Wnt ligands	Restores osteogenesis	[90]
BMSCs	KAT6A	∖	Activates Nrf2/ARE signaling and ameliorates ROS accumulation	[91]
BMSCs	PCAF	CXCL12	Facilitates osteoclastogenesis	[92]
BMSCs; BMMs	HDAC1	JAG1; EEF2	Inhibits osteogenesis via Notch suppression; facilitates osteoclast viability; stimulates inflammation and osteoblast apoptosis	[93, 94, 95]
BMMs	HDAC2	∖	Promotes osteoclastogenesis	[96]
Chondrocytes	HDAC3	Inflammatory factors and p65	Regulates endochondral ossification	[97]
Osteogenic precursor cells; osteoprogenitor cells; BMSCs	HDAC4	HIF‐1α; RUNX2; IGF1	Restrains osteogenic differentiation	[98, 99, 100]
BMSCs	HDAC5	RUNX2	Inhibits osteogenesis	[101, 102]
BMSCs	HDAC6	RUNX2	Inhibits osteogenesis	[103]
BMSCs; BMMs	HDAC7;	NFATc1	Inhibits osteogenesis and osteoclastogenesis	[104]
BMSCs	HDAC8	RUNX2	Inhibits osteogenesis	[105]
BMSC	HDAC9	∖	Enhances osteoblast differentiation	[106]
BMSC	HDAC11	11β‐HSD2	Decreases glucocorticoid antagonists and promotes osteoporosis	[107]
Osteoprogenitor cells	SIRT1	FoxO3; SOD2; OSX	Activates Wnt/β‐catenin signaling and promotes osteogenesis; prevents osteoblast senescence; promotes OSX transactivation	[108, 109, 110]
BMMs	SIRT2	∖	Suppresses osteoclast differentiation	[111]
BMSCs; BMMs	SIRT3	∖	Promotes mitophagy; facilitates mitochondrial biogenesis and impedes osteoclastogenesis	[112]
BMSCs; osteoclasts	SIRT6	ERα	Potentiate angiogenesis and osteoblast differentiation; promotes cell autophagy through AKT–mTOR; facilitates FasL expression and apoptosis	[113, 114, 115]
Osteoblasts	SIRT7	OSX	Promotes OSX transactivation	[110]

Abbreviations: BMSC, bone marrow mesenchymal stem cell; BMM, bone marrow‐derived macrophages; CBP, CREB‐binding protein; EEF2, eukaryotic elongation factor 2; Erα, estrogen receptor α; FoxO3, Forkhead box O3; GCN5, general control nondepressible 5; HDAC, histone deacetylase; HIF‐1α, hypoxia‐inducible factor‐1α; HSD, 11β‐hydroxysteroid dehydrogenase 2; IGF‐1, insulin‐like growth factor 1; JAG1, JAGGED1; KAT, lysine acetyltransferase; MSC, mesenchymal stem cell; Osx, osterix; PCAF, p300/CBP‐associated factor; SIRT, sirtuin; SRSF, Ser/Arg (SR)‐rich splicing factor; SOD2, superoxide dismutase‐2.

### 5.1 Histone Acetylation in Osteoblast and Osteoclast Differentiation

Histone acetylation is modulated by the opposing enzymes, histone acetyltransferases and HDACs. A common understanding of the role of histone acetylation in gene regulation is enhancement in transcription efficiency. This is because it results in alteration in chromatin structure by making N‐terminal of histone protein has electrostatic repulsion with DNA carrying negative charge [116]. Expanding researches have revealed its role in cell proliferation, metabolic reprogramming, and so on, while abnormal level of its catalytic regulators is involved in OP pathogenesis.

Appropriate transcription factors during osteogenesis in skeletal lineage are crucial for the commitment to osteoblast differentiation. As is mentioned previously, some prominent transcription factors in osteogenic differentiation are RUNX2, OSX, SOX9, and so on, whereby their transcription efficacy is closely related to the corresponding acetylation levels.

Expanding evidence has deepen our knowledge of acetylation‐regulated balance between specification of osteoblast and osteoclast. Acetylation is involved in distinct differentiation stages of osteoblast. In BMSCs, KAT2B catalyzes SRSF1 acetylation, which is considered a crucial factor in limb development [87]. In both MSCs and preosteoblasts, KAT8 positively regulates Runx2 and OSX transactivation by targeting their promoter regions [88]. The substrate genes of the acetyltransferase cyclic‐adenosine monophosphate response element‐binding protein (CBP)/p300 mainly includes Runx2, which potentiates Runx2 transcription and promotes osteogenesis [89]. And except for controlling transcription level of certain transcriptional factors, GCN5 directly modulates Wnt ligands via H3K9 acetylation on promoter region, restoring osteogenic differentiation of BMSCs [90]. Other histone methylases are also found to build a link with osteogenesis, for instance, decreased KAT6A is observed in aging OBMSCs, leading to reduction in histone H3 acetylation, that consequently inactivates Nrf2/ARE signaling and causes ROS accumulation [91]. However, gene expression being affected is yet to be discovered. Another mechanism is its involvement in osteoclastogenesis. Specification toward osteoclast originated from macrophages requires stimulating factors, including but not limited to RANKL, CXCL12 expressed by osteocytes and osteogenic cells respectively. A prominent example is PCAF, which acetylates promoter region of CXCL12 in BMSCs, leading to osteoclast differentiation and OP progression [92].

For the role in reactivating transcriptional activity, HDACs‐mediated deacetylation is also proposed to be implicated in osteoblastogenesis and osteoclastogenesis [117]. Opposing histone acetylation and downregulating transactivation, HDAC1 targets histone H3 acetylation at promoter region of JAG1 in BMSCs under mechanical loading, thus attenuating Notch signaling that stimulate osteogenic differentiation [93]. EEF2 is another target gene of HDAC1, whose enhancement in transcription leads to osteoblastogenesis of bone marrow mononuclear cells (BMM) and bone homeostasis in mice [94]. The promotive role of HDAC1 in bone loss is also indicated in diabetic OP, where enhancing HDAC1/3 triggers inflammation and osteoblast apoptosis [95]. Another intriguing example is HDAC4, which may deacetylate hypoxia inducible factor 1 subunit alpha (HIF‐1α) that upregulates vascular endothelial growth factor A (VEGFA), which consequently leads to suppression in osteogenic precursor cells differentiation and aggravate diabetic OP in mice [98]. And such OP induced by glucocorticoids can also be facilitated by HDAC4‐mediated deacetylation of Runx2 and in osteoprogenitor cells [99]. Dysregulation of HDAC4 also accounts for fetal‐derived OP when exposed to prenatal dexamethasone, as upregulated HDAC4 target IGF1 promoter, which impedes osteogenesis in BMSCs [100]. Notably, hypoacetylation Runx2 promoter is also mediated by HDAC5, HDAC6, HDAC8, and SIRT1 in BMSCs [101‐103, 105, 118]. While some other deacetylases, for instance, HDAC9, is revealed to be negatively correlated with osteoblast differentiation through MAPK inactivation [106], and HDAC11 in BMSCs decrease acetylation level of 11β‐hydroxysteroid dehydrogenase 2 (11β‐HSD2) that antagonizes glucocorticoids, leading to impairment in osteogenic differentiation and lower bone mass [107].

Biological function of histone deacetylation is also exhibited in osteoclastogenesis, as HDAC7 downregulates NFATc1, which suppresses β‐catenin activation and leads to impaired bone resorption [104]. Other examples of HDACS‐mediated osteoclastogenesis include HDAC2. HDAC2 in bone marrow macrophages improves Akt activation, leading to phosphorylation of Forkhead box protein O1 (FoxO1) and encourages osteoclastogenesis [96].

It is notable that except for the predominant cells involved in osteogenesis, another evidence indicates HDAC3 also endochondral ossification within chondrocytes via histone deacetylation of genes including inflammatory factors and p65, suppressing NF‐κB and STAT signaling activation to promote osteoclasts activation [97]. In general, histone acetylation is implicated in both cell differentiation via the regulation of crucial transcriptional factors (such as RUNX2 and CXCL12) that mark the commitment to either osteoblast or osteoclast, while defects in its enzymatic regulators (mainly HDACs) result in OP. Overall, HDACs mentioned are crucial to osteoblast or osteoclast differentiation, and are potential therapeutic targets in OP. Further advancements should focus on dysregulated key pathways and catalytic enzymes when bone homeostasis is disrupted.

### 5.2 Nonhistone Acetylation in Osteoblast and Osteoclast Differentiation

Although our understanding of the biological significance of acetylation started off by histone acetylation, expanding researches have been focusing on nonhistone acetylation since the first discovery of the target protein p53 [119]. Different from that of histone acetylation, nonhistone acetylation has a direct regulatory effect on proteins, through alteration in their cellular localization, structural stability, hydrophobicity, and so on [120]. Also, variety in its substrate proteins endows nonhistone acetylation with a wide‐ranging impact on cellular processes.

Nicotinamide adenine dinucleotide (NAD+)‐dependent sirtuins (SIRTs) play a significant role in the balance between acetylation and deacetylation. SIRTs are a group of deacetylases that catalyzes transferring of acetyl moiety to ADP‐ribosyl of NAD+. The SIRT family can be categorized into seven types, with SIRT1, SIRT6, and SIRT7 situated in nucleus [121].

The biological function of SIRT1 spans a wide spectrum, ranging from regulating osteoblast differentiation, osteoblast senescence, to osteocyte necrosis. In osteoprogenitor cells, SIRT1 plays a stimulative role in osteoblastogenesis and osteoprogenitor cell proliferation through deacetylation of FoxO3, which prevents FoxO3 from binding to β‐catenin, and thus activating Wnt/β‐catenin signaling pathway [108]. Another mechanism by which SIRT1 mitigates OP is inhibition of osteoblast senescence. Mechanistically, SOD2 acetylation is opposed by SIRT1, which prevents mitochondrial dysfunction and bone loss [109].

Evidence have shown that both sex‐related and senile OP are correlated to reduction of SIRT3 in BMSCs. Given that SIRT3 is translocated into mitochondria under external cues, biological function of SIRT3 can be concluded as maintaining mitochondrial homeostasis [122] and reserving bone mass. A prominent example is SIRT3. SIRT3 potentiates mitophagy and thus reverses BMSCs senescence under advanced glycation end products induction [123], which is considered an indicator of age‐related bone loss. The impact on mitochondrial function is also demonstrated in BMMs within female mice, as decreased SIRT3 under aging conditions or ovariectomy‐induction impedes mitochondrial biogenesis and facilitates osteoclastogenesis [124], while deletion of SIRT3 by inhibitor LC‐0296 ameliorate reduction in bone mass, induced by insufficient estrogen [112].

Another member of the SIRT family, SIRT6, also strikes a balance between bone formation and bone loss. In bone formation, SIRT6 potentiate angiogenesis and represses ferroptosis in the promotion of osteoblast differentiation in femoral head osteonecrosis [113]. While in age‐related bone loss, decrease in SIRT6 impairs BMSC autophagy through inactivation of AKT–mTOR signaling pathway [114]. In the regulation of bone resorption, deacetylation of ERα locating at Lys171 and Lys299 stabilized Erα, enhancing its transcriptional activity to promote FasL expression and apoptosis in osteoclasts [115].

SIRTs may also have an inhibitory effect on bone loss by affecting osteoclastogenesis. This can be evidenced by SIRT2, as suppression of SIRT2 decreases NFATc1 and c‐Fos expression [111].

With regards to the crucial transcriptional factors, SIRT7 facilitates OSX transactivation via deacetylating K368 on the C‐terminal, followed by depropionylation coupled by SIRT1 [110]. In general, by affecting osteoblast and osteoclast differentiation, osteoprogenitor cell proliferation, mitochondrial biogenesis, BMSC autophagy and other cellular processes, abnormalities in SIRT1‐3, SIRT6, and SIRT7 disrupt balance between bone formation and resorption, thus reducing bone mass and triggering OP. Complete confirmation of the effects that SIRTs exert in bone homeostasis is necessary to prompt potential therapeutic targets for OP.

## 6 Ubiquitination in Bone Homeostasis and OP

Ubiquitination is an evolutionarily conserved PTM that regulate protein degradation reversibly and accurately. The system involves transferring of a 76‐amino acid protein, ubiquitin (Ub), catalyzed by a cascade of enzymes, namely activating (E1), conjugating (E2) and ligating (E3) enzymes. After being activated, transferred, and recognition of substrate protein by E1, E2, and E3 respectively, Ub forms a isopeptide bond between a lysine residue of target protein and the C‐terminal of Ub [125]. Compared with E1 and E2 that involves two and approximately 40 kinds, respectively, E3 is divided into three categories (RING (interesting new gene), HECT (homologous to E6AP C‐terminus), and RBR (RING‐between‐RING) that comprised roughly 600 kinds, which accounts for accurate selection of substrate proteins [126]. Also, as a reversible modification, ubiquitination is opposed by deubiquitination, which is catalyze by dubiquitinating enzymes (DUBs) that remove Ub ligands. The DUBs involve cysteine proteases and metalloproteases, while the former one comprises Ub‐specific protease (USP), K48 polyubiquitin‐specific MINDY domain families, Ub C‐terminal hydrolase (UCH), ovarian tumor (OUT), Machado‐Josephin domain (MJD), and the zinc finger with UFM1‐specific domain [127].

**FIGURE 3:**
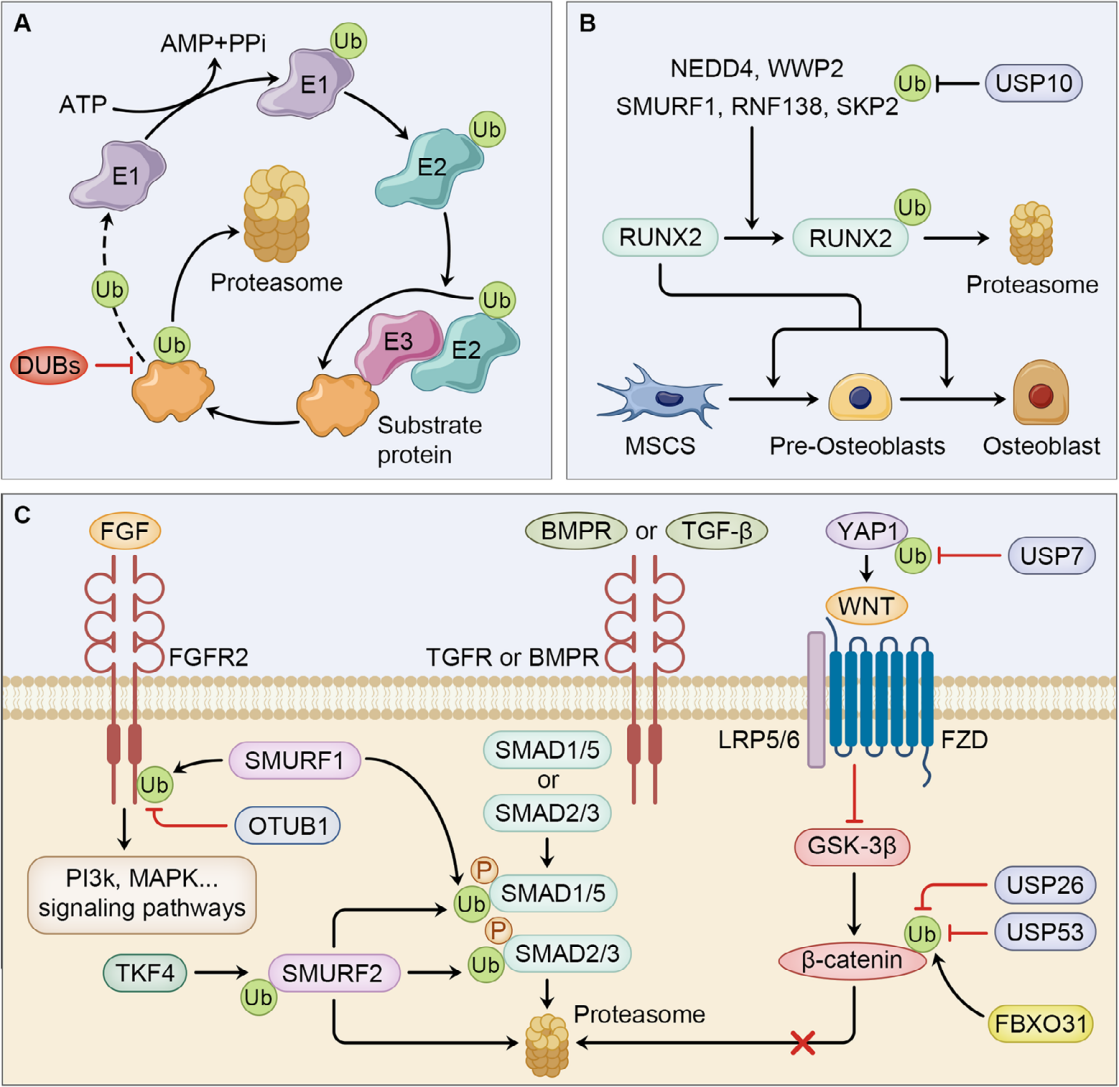
Ubiquitination in bone homeostasis and osteoporosis. (A) Ubiquitination is a reversible process mediated by a cascade of enzymes, including E1‐activating, E2‐conjugating, and E3‐ligating enzymes, while the Ub can be removed by DUBs and thus preventing protein from proteasome degradation. (B) Several E3 ligases, including NEDD4, WWP2, SMURF1, RNF138, and SKP2, target RUNX2 for ubiquitination and degradation, thus suppressing commitment to osteoblast in BMSCs, and USP10 also inhibits osteogenesis through deubiquitination of SKP2. (C) ubiquitin signaling is also involved in bone homeostasis and osteoblast differentiation. FGF signaling is inhibited or promoted through the regulation of SMURF1 and OTUB1 respectively. SMURF 1 is also involved in the regulation of BMP signaling pathway through ubiquitinating SMAD1/5, while SMURF2, which can ubiquitinated by TKF4, targets SMAD proteins for modification and degradation, leading to BMP signaling deactivation. In the regulation of WNT signaling, osteoblast differentiation is promoted through stabilization of YAP1 and β‐Catenin mediated by USP7, USP26, and USP53 respectively, while β‐Catenin ubiquitinated by FBXO31.

Ubiquitination is broadly implicated in cellular processes, including but not limited to cell autophagy, DNA repair, and apoptosis. Secondary to phosphorylation, the high Ub level implicated in signal transduction indicates their indispensable role in mediating signaling networks though degradation of substrate proteins, alteration of protein activity, or changes in protein interaction [128]. Growing evidence has expanded our knowledge of biological role of ubiquitination and deubiquitination in bone homeostasis and osteoarthritis progression. In this section, we discuss the updating researches concerning the control over osteogenesis and correlated cellular processes affecting cell function regulated by ubiquitination in bone development and OP (Figure 3).

### 6.1 Ubiquitination in Cell Differentiation

In bone development, particular transcriptional factors and signaling pathways are required in commitment to osteoblasts or osteoclasts, which are strictly controlled by ubiquitination and deubiquitination. Dysfunction of the correlated enzymes leads to aberrant number in osteoblasts and osteoclasts, resulting in decreased bone mass and OP.

A transcriptional factor included in osteoblast differentiation is Runx2, which potentiate commitment to osteoblast differentiation and is mostly regulated by ubiquitination and deubiquitination. In M‐BMSCs, Runx2 is targeted for poly‐ubiquitination and degradation by the E3 ligase, NEDD4, while upregulation of Nedd4 represses osteogenic differentiation [129]. Notably, Runx2 is also mono‐ubiquitinated by WWP2, a member of NEDD4 family, which potentiate its transcriptional activity and osteoblastic differentiation, rather than degrades it [130]. NEDD4 also ubiquitinates Runx1, another member of the Runt domain transcription factors that activates pathways like WNT/β‐Catenin to impede bone loss, thus decreasing BMSCs osteogenic commitment [131, 132]. Besides, Smurf1, RNF138, and S‐phase kinase‐associated protein 2 (SKP2) are also linked to degradation of Runx2 and inhibition of bone development [133]. Notably, the SKP2 is deubiquitinated by USP10, leading to suppressed osteogenic differentiation in BMSCs [134].

The transcriptional factor, Msh homeobox 1 (MSX1), is required in early morphogenesis, as well as osteogenic differentiation. A newly identified DUB stabilizing MSX1 is USP11, which enhances ALP activity and osteogenic capacity [135].

The signaling pathways involved is FGF signaling pathway, where the FGFRs are bounded to FGFs to activate downstream signaling pathways in the regulation of bone development. Usually, FGFRs are the substrate protein in the control of bone homeostasis by ubiquitination or deubiquitination. OTUB1 removes Ub from FGFR2 that catalyzed by the E3 ligase SMURF1, and overexpression of OTUB1 in OP mouse model leads to restoration of bone mass [136].

The BMP signaling has been indicated to be indispensable in osteogenesis, as dysregulation of BMPs and BMPRs leads to abnormal deficiencies in bone development and OP. The salient ubiquitinating enzymes regulating BMP signaling are the Smad ubiquitination regulatory factor (Smurf) 1 and Smurf2. Previous studies have shown that both Smurf1 and Smurf2 have an inhibitory role in osteogenesis through the ubiquitination of Smad1/5 and Smad2/3, respectively [137, 138], while new evidence shows that Smurf2 also targets Smad1/5/8 for degradation, leading to decreased skeletal size and increased bone mass in mice [139]. Notably, as a substrate protein of TNF receptor‐associated factor 4 (TRAF4), Smurf2 can be degraded in MSCs to enhance osteoblast differentiation [140].

Wnt/β‐catenin signaling is another crucial pathway that activates preosteoblast committing to osteoblast by inducing OSX expression [3]. The Wnt/β‐catenin pathway can be activated to ameliorate OP by Yes1‐associated transcriptional regulator (YAP1), which is deubiquitinated and stabilized by Ub specific peptidase 7 (USP7) in MSCs [141]. Also, in the regulation of Wnt/β‐catenin pathway, a newly identified DUB in bone formation, USP26, exerts a pro‐osteoblastic and antiosteoclastic effect through the stabilization of β‐catenin in MSCs and IκBα in BMMs [142]. In addition to mediate β‐catenin directly, USP53 binding with FBXO31 further stabilizes β‐catenin in promotion of Wnt/β‐catenin activation in BMSCs and bone regeneration [143].

However, the role of USP7 in osteoclast differentiation remains unclear and context dependent. While in BMMs, USP7 exerts inhibitory impact on bone resorption through suppression of osteoclastogenesis. It is suggested that by removing K63‐polyubiquitin chain on TRAF6 and STING, USP7 suppresses NF‐κB and MAPK signaling and activates STING pathway, leading to inhibition of osteoclastogenesis‐related gene expression and induction of IFN‐β that impede bone resorption [144]. The biological role of USP7 in bone formation differs in CD14+ peripheral blood mononuclear cells, as it targets HMGB1 for deubiquitination, which activates NF‐κB signaling pathway and facilitate osteoclast differentiation [145]. Also, in the control over osteoclast differentiation, Ub C‐terminal hydrolase 1 (UCHL1) directly removes Ub from PDZ‐binding motif (TAZ) protein, which stabilizes TAZ that diminishes the binding capacity of NFATC1, resulting in impairment in osteoclastogenesis‐related gene transcription [146]. The degradation of another pro‐osteoclastic factor, estrogen receptor‐related receptor alpha (ERRα) is catalyzed by STUB1, which ubiquitinates ERRα at K51 and K68, thus inhibiting osteoclast differentiation and bone loss, suggesting STUB1 as a potential target for postmenopausal OP [147]. In contrast, the antiosteoclastic factor, nuclear factor‐erythroid 2‐related factor 2 (Nfr2), is ubiquitinated by the E3 ligase, Hrd1. And inhibiting Hrd1 in BMMs mitigates production of ROS and inflammatory cytokines, leading to amelioration of ovariectomy‐induced OP in mice [148].

### 6.2 Ubiquitination in Cell Function

Except for having impact on differentiation of osteoblast, ubiquitination and deubiquitination may also target proteins implicated in cellular processes, including but not limited to mitochondrial metabolism, mitophagy, inflammasome activation, cell senescence, apoptosis, and so on.

For example, in osteoclast, *BRCA1‐associated protein 1* (BAP1)‐mediated deubiquitination of H2AK119ub1 on Slc7a11 promoter, which is a cystine reporter that enhances glutathione synthesis and impairs TCA cycle, is essential for osteoclast function [149]. Other evidence points out the correlation between mitochondrial dysfunction and bone homeostasis, as degradation of mitochondrial membrane proteins ubiquitinated by E3 ligase, Parkin, leads to mitophagy that prevents ROS accumulation and osteoblast apoptosis [150, 151]. Studies have shown that NLRM3 inflammasome activation also has an established link to post OP, while USP1 interacts with the E3 ligase, TRAF6, which suppresses NF‐κB signaling, thus inhibiting NLRP3‐induced pyroptosis in osteoblast [152]. An E2 conjugating enzymes with an established correlation to cell senescence in bone formation is Ub‐conjugating enzyme E2 E3 (UBE2E3), as downregulation of UBE2E3 within aging bone marrow leads to decrease of Nrf2 in nucleus as well as its expression, which accelerates BMSCs senescence and impeding osteogenesis [153]. Besides, absence of estrogen causes cellular senescence through downregulation of USP10, which deubiquitinates p53 and stimulates bone formation, suggesting USP10 as a positive regulator of bone development [154]. Another cellular process involved is apoptosis induced by p53. P53 is activated under exposure to dexamethasone in osteoblastic cells, whereby USP14 contributes to stabilization of p53 that leads to impaired osteogenic differentiation and OP suffering from GC treatment [155]. Overall, targeting ubiquitinating and deubiquitinating enzymes are considered as potential therapeutics in bone disorders. Further research should complete understanding of the role that these catalytic enzymes have in OP development.

## 7 Sumoylation

Similar to ubiquitination, Sumoylation is a similar form of PTM that mediates conjugation of small Ub‐like modifier (SUMO) to a substrate protein. The SUMOs, including five isoforms, have been identified in mammalian cells, while SUMO1‐3 are presented in human cells. SUMO2 and SUMO3 are highly homologous and contribute to formation of a poly‐SUMO chain [156]. Different from ubiquitination that undergoes activation, conjugation, and ligation catalyzed by E1, E2, and E3 enzymes, additional maturation of SUMO is required for a SUMO to be tagged on lysine residue of the target protein. Mediated by sentrin‐specific precursor protease 1 (SENP), the C‐terminal di‐glycine residue of SUMO is exposed, which is further activated, transferred, and connected to substrate protein by SUMO‐activating enzyme E1 (SAE1/SAE2), SUMO‐conjugating enzyme E2 (UBC9), and SUMO E3 ligase respectively. Notably, ligation of SUMO is not essential in SUMO cycle [157, 158]. The process of SUMOylation is also opposed by de‐SUMOylation, catalyzed by the six SUMO‐specific proteases (SENPs) identified in human tissues [159]. Among the SENP family, SENP3 has high affinity for de‐SUMOylation of SUMO2/3. Unlike ubiquitination that mediates proteasome degradation of the substrate protein, the direct impact of SUMOylation on target protein involves alteration or creation of new sites for protein–protein interaction, cellular location of protein, protein transcriptional activity, protein stability, and so on. Given that over 6000 proteins can be tagged by SUMOs, the biological functions of SUMOylation are involved in autophagy, apoptosis, DNA damage, and so on [160, 161].

A prominent effect of SUMOylation lies in the suppression of transcriptional activity, where the SUMO protease, SENP3, has attracted most attention in the regulation of osteoblast and osteoclast differentiation in OP development. However, its biological function in bone homeostasis seems to be conditional and context‐based. A study focusing on glucocorticoid‐induced OP in BM‐MSCs shows that overexpression of SENP3 impedes osteogenic differentiation and potentiate osteogenic differentiation by reversing SUMO2/3 modification of PPARγ2 at K107, resulting in imbalanced proportion between adipocytes and osteoblast [162].

The result contradicts with the findings reported by Zhang et al. and Wang et al., both of which shows an osteoprotective effect of SENP3 in OP. In BMMs, Zhang et al. observed that impairment in SENP3 leads to osteoclastogenesis. Mechanistically, SENP3 targets interferon regulatory factor 8 (IRF8) at K310 for de‐SUMOylation, which inhibits NFATc1 expression and impedes osteoclast differentiation [163]. Also, Wang et al. [164] show that concerning type II diabetes‐induced OP, SENP3‐mediated de‐SUMOylation of SUMO2/3 on HIF‐1α stabilizes HIF‐1α, leading to potentiation of PPAR‐γ transcription that causes osteoclastogenesis suppression in BMMs. The discrepancy in the role of SENP3 may be explained as follows: first, the impact of SUMOylation on substrate protein remains complicated, as it may changes protein–protein interaction or increase protein stability; second, complicate signaling crosstalks involved in osteogenesis and osteoclastogenesis may account for diversity in the response to different cellular stress within BMSCs and BMMs.

**FIGURE 4:**
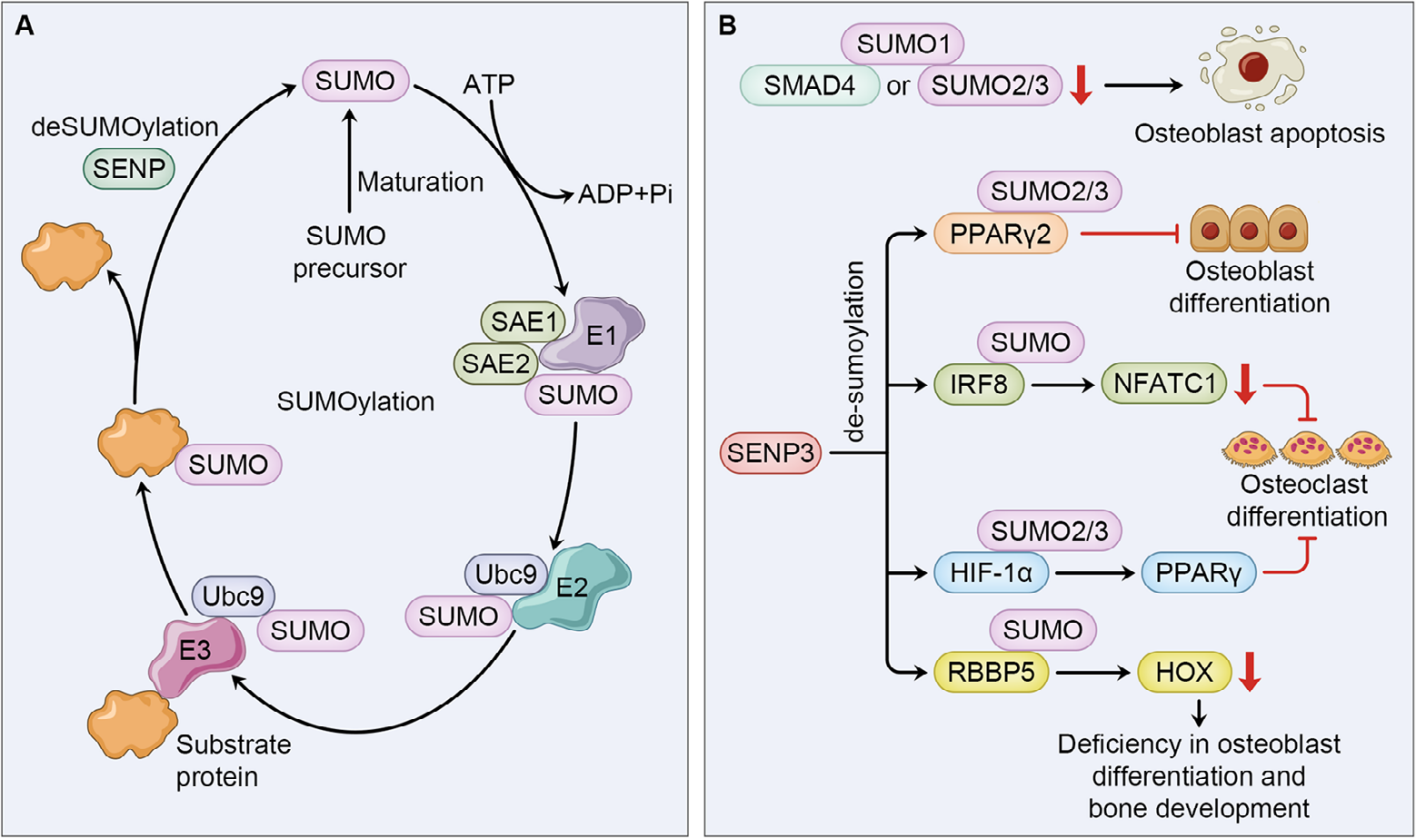
SUMOylation in bone homeostasis and osteoporosis. (A) SUMOylation is a reversible PTM, where SUMO is linked to substrate protein under the regulation of SAE1, SAE2, E2 UBC9, and E3 ligases, and removed by SENPs. (B) Decreased SUMOylation of SMAD4 promotes osteoblast apoptosis. And de‐SUMOylation of PPARγ2, IRF8, HIF‐1α mediated by SENP3 impairs differentiation of both osteoblast and osteoclast, while removing SUMO from RBBP5 mediated by SENP3 leads to reduced HOX transcription and inhibition of osteogenesis.

Except for directly affecting transcriptional activity in osteogenesis and osteoclastogenesis, SUMOylation may influence bone homeostasis through regulating cell death, as induced by oxidative stress, deficiency in SUMO1 or SUMO2/3 tagged on SMAD4 leads to osteoblast apoptosis, suggesting significance of SUMOylation of SMAD4 in DNA damage and osteoblast viability [165]. Additionally, SUMOylation is implicated in regulation of another form of PTM, methylation. This is best illustrated by SENP3, which de‐SUMOylates RbBP5, a component of a histone methyltransferase complex, leading to deficiency in H3K4 methylation and HOX genes transcription that encodes transcriptional factors concerning MSC differentiation and skeleton development [166, 167] (Figure 4).

## 8 Glycosylation

**FIGURE 5:**
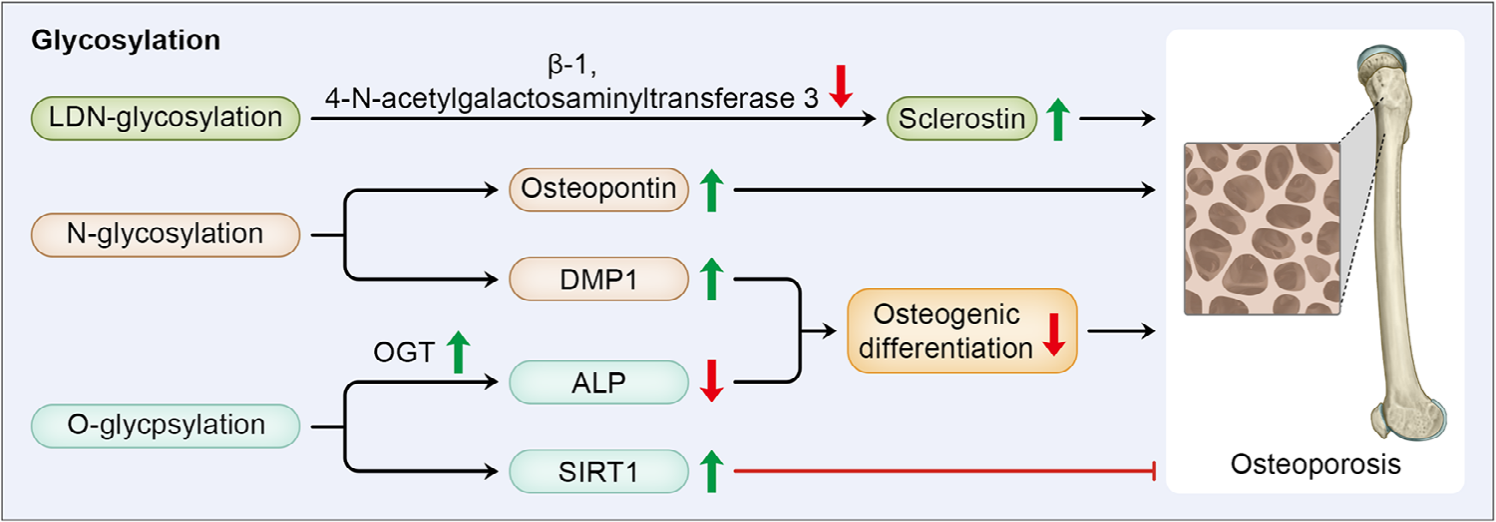
Glycosylation is involved in osteoporosis mainly through the regulation of osteoblast differentiation and expression of sclerostin and osteopontin. Catalyzed by beta‐1,4‐N‐acetylgalactosaminyltransferase 3, LDN‐glycosylation facilitates sclerotin degradation, and thus inhibiting osteoporosis. N‐glycosylation of osteopontin leads to bone mass loss and osteoporosis. In the regulation of osteogenic differentiation, N‐glycosylation of DMP1 and O‐glycosylation of ALP impede osteogenesis, thus exerting a promotive effect in osteoporosis development. On the contrary, O‐glycosylation of SIRT1 shows an osteoprotective effect through the further deacetylation of RANKL.

Glycosylation is the most common and abundant form of PTM, as almost 50% of proteins are glycosylated. Glycosylation forms when single sugars or glycans are covalently bound to the residues of substrate proteins. Based on the protein‐sugar linkage, glycosylation can be categorized into N‐glycosylation, O‐glycosylation, C‐glycosylation, P‐glycosylation, with N‐glycosylation and O‐glycosylation being mostly discussed [168]. With large properties in substrate proteins and diversity in forms of modification, glycosylation plays a multifaceted role in cellular function, such as signaling transduction, protein stability, and intercellular interaction [169]. In the regulation of bone formation, glycosylation mainly affects osteoblast differentiation and modifies osteopontin that potentiate NF‐κB pathway. In this section, we will summarize the effects of glycosylation on bone homeostasis and emphasize its potential role in cell differentiation (Figure 5).

### 8.1 LDN‐Glycosylation

Sclerostin is an glycoprotein derived from osteoblasts and osteocytes, which is negatively correlated with bone mass by suppressing Wnt/β‐catenin signaling pathway and osteoblast function [170] Transferring of N‐acetylgalactosamine into N‐acetylglucosaminebeta‐benzyl catalyzed by beta‐1,4‐N‐acetylgalactosaminyltransferase 3 induces LacdiNAc (LDN)‐glycosylation of sclerostin, which prompts it to undergo degradation, thus preventing OP progression [171]. As evidence has indicated a protective effect of sclerostin antibodies in bone formation and bone strength [170], B4GALNT3 is suggested a bone‐specific therapeutic target in OP.

### 8.2 N‐Glycosylation

N‐glycosilation refers to the relationship between GlcNAc and amide nitrogen atom of asparagine at a specific amino acid sequence combined by N‐glycosidic [172]. PTM is believed to play a vital role in direction of differentiation for the constant protein sequencing within the cells. While N‐glycosylation is implicated in the determination of differential fate in BMCs, explained by discrepancy in glycosylation of tissue nonspecific ALP (TNAP). High level of TNAP activity potentiates adipogenesis, while a higher demand for TNAP activity activates commitment to osteoblast in ADSCs. Differences in glycosylation levels of TNAP in BMCs from different tissue corresponds to their differential fate, suggesting a regulatory effect of glycosylation pattern in cellular differentiation [173].

Another example implies the role of N‐glycosylation in mediating cell function, as modification of osteopontin, another extracellular matrix protein encoded within osteoblasts, has been identified to activate NF‐κB signaling pathway in both osteoblast and osteoclast, thus causing loss of bone mass [174], suggesting targeting osteopontin N‐glycosylation in both osteoblast and osteoclast for OP treatment. Except for affecting cellular function of mature osteoblast, dysregulation of N‐glycosylation is also involved in craniofacial development, as N‐glycosylation of dentin matrix protein 1 (DMP1) impedes osteogenic differentiation to suppress cranial suture fusion, while mutation in its binding site exhibit premature suture fusion in mice [175].

### 8.3 O‐Glycosylation

O‐glycosylation is established between the hydroxyl groups on sugar isomer and specific residues on targeted glycoproteins, including serine (Ser), threonine (Thr), tyrosine (Tyr), or hydroxylysine (Hyl). According to the monosaccharide potentiating O‐glycosylation, it can be divided as Fuc, Glc, Man, and GlcNAc O‐glycosylation [172].

O‐glycosylation exerts its biological function in bone homeostasis through direct regulation of differentiation of skeletal lineage, as well as enzymes involved in other PTMs. This is best illustrated by *O*‐acetylglucosamine (*O*‐GlcNAc) modification of ALP. Situated on the cell membrane, TNAP hydrolyzes pyrophosphate to inorganic phosphate in extracellular space [176]. Serving as the biomarker for bone marrow stromal stem cell subpopulation to differentiate into osteogenic lineages, TNAP in bone progenitor cells regulates mitochondrial function and ATP levels to strengthen bone mineralization and trabecular bone formation [177]. Activity of O‐GlvNAcylated ALP decreases under regulation of O‐linked N‐acetylglucosamine transferase, which impairs osteogenic differentiation in periodontal ligament cells [178]. However, O‐GlyNAcylation of SIRT1 shows a protective effect in bone homeostasis. As mentioned previously, SIRT1 has an osteoprotective role in osteoblast, while its catalytic activity is increased by *O*‐GlcNAc modification at N346R, thereby deacetylating RANKL and promoting osteoblast proliferation [179]. Therefore, the O‐glycosylation on SIRT1 is suggested a targeted therapy for attenuating OP.

## 9 Other Types of PTMs in Bone Homeostasis and OP

### 9.1 ADP‐Ribosylation

ADP‐ribosylation is a reversible process where the ADP‐ribose (ADPR) is connected to substrate protein. According to the modifying group, ADP‐ribosylation is categorized as mono ADP‐ribosylation (or MARylation) and poly ADP‐ribosylation (or PARylation). The dynamical process is regulated by the writer protein, ADP‐ribosyl transferase (ADPRT or ART), which can be further divided as the cholera toxin‐like ADP‐ribosyl transferases (ARTCs) and the diphtheria toxin‐like ADP‐ribosyl transferases (ARTDs). Five and seventeen kinds of ARTCs and ARTDs have been identified in human cells respectively, with the 17 ARTDs (also known as PARPs) catalyzing transferring of poly ADP‐ribose. Notably, ARTD3, 4, 6, 10, 14–16 only contribute to MARylation. Similar to other forms of PTM, ADP‐ribosylation is erased by two families of hydrolase, the ADP‐ribosyl‐acceptor hydrolases and the macrodomain‐containing enzymes. ADP‐ribosylation is involved in a wide range of cellular processes, including but not limited to DNA repair, energy metabolism, signaling transduction, and so on [180].

**FIGURE 6:**
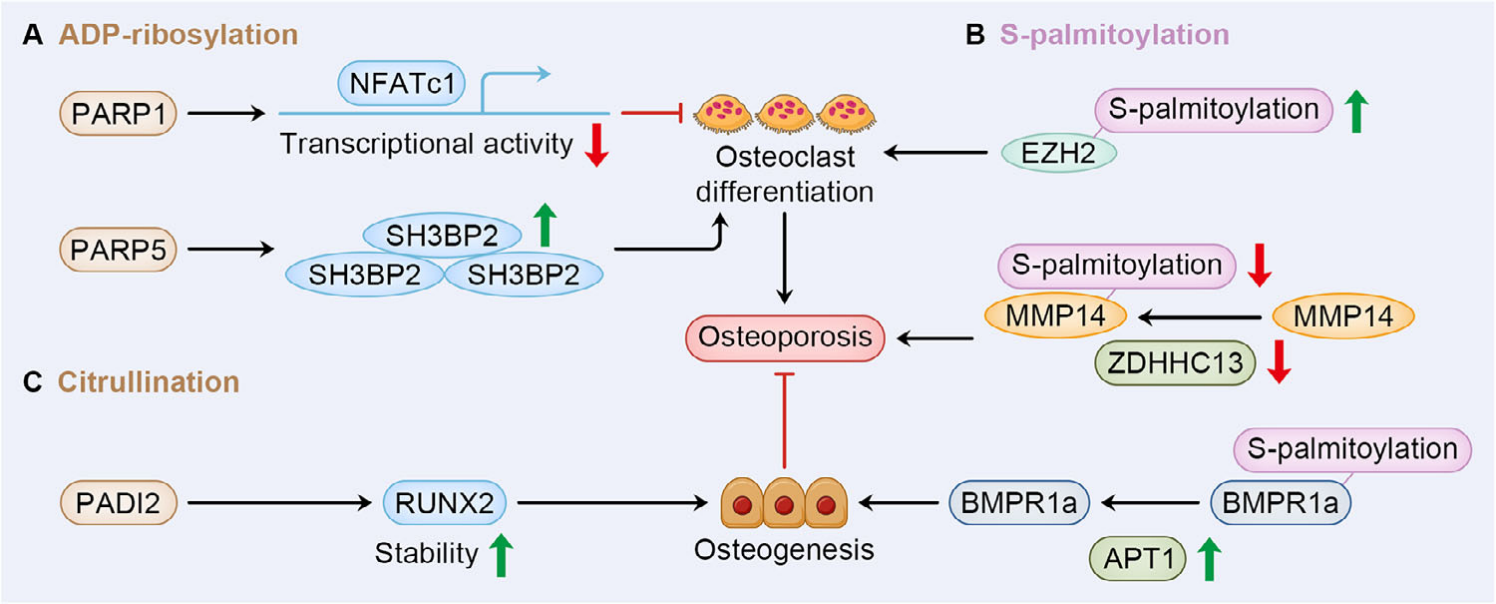
ADP‐ribosylation, S‐palmitoylation, and citrullination in bone homeostasis and osteoporosis. (A) Several catalytic regulators of ADP‐ribosylation are involved in the regulation of osteoclast differentiation. PARP1 has an inhibitory effect on osteoclastogenesis via the suppression of NFATc1 transcription. PARP5 has an opposite effect on osteoclast differentiation through the accumulation of SH3BP2. (B) S‐palmitoylation affects both osteoclast and osteoblast differentiation in the regulation of osteoporosis. In the regulation of osteoclastogenesis, palmitoylation of EZH2 facilitates osteoclast differentiation, thus promoting osteoporosis development. Through the palmitoylation of MMP14, inhibition of the catalytic regulator, ZDHHC13, leads to MMP14 relocation and promotes osteoporosis. In the regulation of osteoblastogenesis, enhanced APT1 diminishes BMPR1a palmitoylation, thus activating osteogenesis and restraining osteoporosis.

ADP‐ribosylation has been considered a potential target for OP treatment, as dysfunction of PARP in preosteoblastic cells has been shown to encourage osteoblast differentiation and enhance cell viability [181], while suppression of PARG impedes osteoclast differentiation [181]. Among the catalytic regulators of ADP‐ribosylation, PARP1 is mostly researched regarding its function in both osteoblast and osteoclast differentiation, which can be concluded as osteoprotective. In regulation of osteoclastogenesis, PARP1 mediates modification on H2B at serine7 and blocks occupancy at NFATc1 promoter, thus inhibiting NFATc1 transcriptional activity [182]. Another mechanism by which PARP1 suppresses osteoclast differentiation is modification at IL‐1β, which also impedes NFATc1 expression that positively correlated with osteoclastogenesis [183]. Another PARP that draws the attention in bone homeostasis is PARP5, which exerts an opposite effect on osteoclastogenesis, as inhibition of PARP5 leads to accumulation in SH3 domain‐binding protein 2 (SH3BP2) that positively regulates osteoclastogenesis‐related signaling [184] (Figure 6A).

### 9.2 S‐Palmitoylation

S‐palmitoylation is a lipid PTM reversibly mediated by zinc DHHC‐type containing (ZDHHC). Under the catalyzation of ZHDDC enzyme family, palmitate, the palmitic acid converted from fatty acids, is linked with Cys residue connected by a thioester bond [185]. Depalmitoylation is regulated by PPT1/2, acyl‐protein thioeterases (APT1/2), and alpha/beta hydrolase domain‐containing protein 17A/B/C (ABHD17A/B/C) [186]. Based on its prominent effect of increase in hydrophobicity on targeted protein, palmitoylation could have a profound influence on protein function, such as protein–protein interaction, protein stability, protein subcellar trafficking, thus altering a wide range of cellular processes [187]. Also, dysregulation of palmitoylation and depalmitoylation has emerged as a potential target in OP progression [188].

Depending on the substrate protein, palmitoylation affect both osteoblast and osteoclast differentiation. Expanding evidence has indicated its role in osteogenesis, as supported by differentiated expression of different subtypes of ZDHHC in mouse calvarial osteoblasts [188]. The impact of decrease in types of ZDHHCs (ZDHHC1, ZDHHC2, and ZDHHC12) that causes lipotoxicity is also exhibited by upregulation of these enzymes induced by 1,25(OH)_2_D_3_ within osteoblast treated by lipid [189]. In regulation of osteoclastogenesis, inhibition of STAT3 and EZH2 palmitoylation within BMMs leads to impaired RANKL‐induced osteoclast differentiation as exhibited by decrease in c‐Fos and NFARc1 expression [190]. The result corresponds with the finding that mutation in Zdhhc13 is positively correlated with loss of bone mass and OP in mice, while deficiency in palmitoylation of membrane type 1‐matrix metalloproteinase (MMP14) mediated by ZDHHC13 redistributes MMP14 to perinuclear region and impairs angiogenesis through reduction in VEGF [191]. Dysregulation of depalmitoylation also affects bone formation. While in BMSCs, the APT1 is considered a positive regulator of osteogenesis‐related signaling, as in senile OP by reversing BMPR1a palmitoylation and altering BMPRa transport and localization, thereby activating BMP/Smad pathway that potentiate osteogenesis [187] (Figure 6B).

### 9.3 Citrullination

Citrullination refers to a form of PTM refers to the conversion of peptidyl‐arginine into peptidyl‐citrulline mediated by peptidylarginine deiminases (PADIs). Biological function of citrullination is involved in protein–protein interaction, protein distribution for its basic effect of alteration in charge. Though there are five kinds of PADI isozymes existing in mammal cells [192], less is known about the role of citrullination in bone homeostasis and OP. But a study concerning cleidocranial dysplasia points out the potential effect of citrullination in osteoblast differentiation, as PADI2 targets RUNX2 for citrullination, which stabilized RUNX2 that promotes skeletogenesis [193] (Figure 6C).

## 10 Perspectives

A coordinated balance between bone formation and bone resorption is essential for bone regeneration, while dysregulation of bone homeostasis gives rise to lower bone mass and susceptibility of OP. In the regulation of bone homeostasis, PTM dynamically regulates the abundance and functions of proteins implicated in signaling networks, thereby having a profound influence on osteogenesis and affecting the balance between osteoblasts and osteoclasts. The biological role of each PTM is multifaceted and sophisticated, depending on the substrate protein, as well as the certain cell type. This is best illustrated the contradictory effect of SENP3 on bone formation, as SENP3 exerts inhibitory impact on bone formation and bone resorption through de‐SUMOylation of PPARγ2 and IRF8 in BMSCs and BMMs, respectively. In addition, the complexity of the biological role of PTM can be explained by the crosstalk among each type of it. For example, phosphorylated β‐catenin can be further ubiquitinated for degradation, leading to suppression of Wnt signaling pathway and impairment in osteoblast differentiation. Another intriguing example is the relationship between glycosylation and acetylation, as O‐GlyNAcylation of SIRT1 enhances its acetylating activity and promote osteoblastogenesis. Except for acting sequentially on substrate protein or enhancing catalytic activity of other enzymes, the crosstalk among different PTMs may exhibit as mutual exclusion, as ubiquitination of Runx3 is inhibited by its competitor lysine acetylation [194]. Such PTM crosstalk also accounts for the discrepancy of seemingly paradoxical results about the effect of some catalytic regulators. It is notable that though this review summarizes the latest findings concerning functions of main types of PTM in osteogenesis and OP development, some other types of PTM are not presented in this review, such as glycation and lysine benzoylation.

Since the substrate protein of each PTM participate in OP pathogenesis, drug candidate targeting catalytic regulators of the corresponding modification is considered potential treatment for OP. Through intervention in PTMs of proteins, it is possible to regulate the abundance or function of certain proteins, thereby regulating OP‐relevant proteins. A study has shown that 2‐bromopalmitic acid that serves as an inhibitor of S‐palmitoylation potentially inhibits osteoclast differentiation, which mitigates OP in ovariectomized mice [190]. A novel oleanane triterpenoid compound, RTA‐048, impairs osteoclastogenesis by blocking TRAF6‐mediated STING ubiquitination, which causes inhibition in NF‐κB signaling [195]. Despite the fact that clinical actuality is calling for novel therapeutic candidates based on PTMs, however, the gap between therapeutic actuality and bench findings still exists, as relevant preclinical animal experiments and clinical trials are lacking. Also, the complexity and extensiveness of each PTM implicated in numerous cellular processes have not been fully studies, which indicated a possibility of affecting normal cells within bone tissue when targeting some specific catalytic regulators.

Therefore, our knowledge of some catalytic regulators involved in PTMs is yet to be expanded, which relies on cutting‐edge technologies to identify new catalytic mediators of PTMs, as well as novel PTM types. Identification of the biological role of PTMs in bone biology and OP is still based on single cases and conventional biochemical techniques, for instance, immunoblotting, coupled enzyme reactions, nuclear magnetic resonance, and so on. Another commonly used technique for protein PTM researches is mass spectrometry (MS)‐based analysis, which allows for the identification of both PTM type and modification site [196]. However, low concentration of PTM of interest limits the precise quantification of MS, while alterations in the structure of modified residues also exerts challenges on current biochemical tools to identify [197]. Therefore, novel techniques for proteomics are required for higher efficiency and accuracy in understanding biological role of PTMs. Emerging researches have suggested some novel methodologies for higher accuracy and sensitivity in PTMs identification, for example, utilizing biorthogonal labeling and chemical probes to enrich protein PTMs of interest is demonstrated effective [198, 199, 200]. It is believed that application of new proteomic technology will deepen our understanding of involvement of PTMs in bone homeostasis, bringing novel therapeutic targets for OP treatment. Also, for further confirmation of PTMs’ role in bone homeostasis and feasibility of therapeutic targets in clinical trials, it requires strict methods of cell isolation from fresh tissue. With the application of single‐cell sequencing, it is possible to prevent certain cell population degeneration, identify novel subpopulation, and deepen our understanding of relationship between PTMs and osteogenic differentiation.

## Author Contributions

Y. S. L. conceived of the study and revised the manuscript. Y. Z. L. contributed to writing the manuscript. S. D. J. and H. Z. L. contributed to figures and revision discussions. H. F. J. and G. Y. contributed to idea discussion. Y. M. Y. and B. Z. J. contributed to data collection. All authors read and approved the final manuscript.

## Ethics Statement

The authors have nothing to report.

## Conflicts of Interest

The authors declare no conflicts of interest.

## Data Availability

The authors have nothing to report.
